# Initial presenting symptoms, comorbidities and severity of COVID-19 patients during the second wave of epidemic in Myanmar

**DOI:** 10.1186/s41182-021-00353-9

**Published:** 2021-08-06

**Authors:** Ye Minn Htun, Tun Tun Win, Aung Aung, Thant Zin Latt, Yan Naung Phyo, Thet Min Tun, Nyan Sint Htun, Kyaw Myo Tun, Khin Aung Htun

**Affiliations:** 1 Department of Prevention and Research Development of Hepatitis, AIDS and Other Viral Diseases, Health and Disease Control Unit, Nay Pyi Taw, 15011 Myanmar; 2 Department of Preventive and Social Medicine, Defence Services Medical Academy, Mingalardon, Yangon, Myanmar; 3 Department of Research and Development, Defence Services Medical School, Hmawbi, Yangon, Myanmar; 4 Outpatient Department, No. 3 Military Hospital (100 Bedded), Hlegu, Yangon, Myanmar; 5 Department of Surgery, Defence Services Medical Academy, Mingalardon, Yangon, Myanmar

**Keywords:** Comorbidities, COVID-19, Initial presenting symptoms, Pneumonia, Severity

## Abstract

**Background:**

Coronavirus disease 2019 (COVID-19) caused by a highly contagious virus called severe acute respiratory syndrome coronavirus 2 emerged in China at the end of 2019 and became a major threat to health around the world. The health experts are still learning more about the detailed knowledge of the natural course and the severity of COVID-19. The study aimed to assess the prevalence and association of severity of disease with demographic characteristics, initial presenting symptoms, and comorbidities among COVID-19 patients in treatment centers, Myanmar.

**Methods:**

A cross-sectional study was conducted at Hmawbi and Indine treatment centers, Yangon Region, Myanmar, from November to December 2020. Data were collected by using standardized case report forms and then, a total of 222 confirmed COVID-19 inpatients were included in this study. The odds ratio with a 95% confidence interval (CI) was used as a measure of association and the independent associated factors for severity of disease were investigated using logistic regression analysis.

**Results:**

In total, 81.5% were symptomatic patients and of these, the most common presenting symptoms were fever 54.1%, loss of smell 50.3%, and cough 30.9%. Among 37.8% of COVID-19 patients with comorbidities, the most common comorbidities were hypertension 58.3%, diabetes mellitus 29.8%, and heart diseases 26.2%, respectively. As a severity, 20.7% of patients had signs of severe pneumonia. The associated factors of severe pneumonia were aged 60 years and older [Adjusted odds ratio (AOR) = 2.88, 95% CI 1.14–7.29]**,** overweight or obesity (AOR: 3.87, 95%CI 1.80–8.33), and current smoking (AOR: 6.74, 95% CI 2.72–16.75).

**Conclusions:**

In this study, one-fifth of the patients developed severe pneumonia. The COVID-19 patients who were aged 60 years and older, overweight or obesity, and current smokers should be monitored carefully during the course of treatment to reduce the disease severity.

## Background

In early December 2019, the first pneumonia cases of unknown origin were identified in Wuhan, the capital city of Hubei province [1]. Since then, there has been a rapid spread of the virus in China and other countries, leading to a global public health problem. On 30th January 2020, the World Health Organization (WHO) has announced coronavirus disease 2019 (COVID-19) as a public health emergency of international concern [2]. After increasing the rapid spread of confirmed cases and continuing the risk of further global spread, the WHO declared COVID-19 as a pandemic on 11th March 2020. Since then, Europe and America had become the epicenter of the pandemic with more confirmed cases and deaths than the rest of the world [3]. As of 14th March 2021, the total number of confirmed cases was 119 million with approximately 2.6 million deaths in 219 countries and territories around the world [4].

The COVID-19 was a highly contagious infectious disease caused by severe acute respiratory syndrome coronavirus 2 (SARS-CoV-2), belonging to an enveloped *β-coronavirus* genus [5, 6]. Host cell binding and entry were mediated by the spike (S) protein of the viral envelope. The S1 subunit of the S protein contained the receptor-binding domain that bound to the peptidase domain of the angiotensin-converting enzyme 2 (ACE2) receptor. SARS-CoV-2 had a greater affinity for the upper respiratory tract. Thus it could infect the upper respiratory tract and conducted airways easily [7, 8].

The SARS-CoV-2 virus was primarily transmitted from infected people to others who were in close contact through respiratory droplets, by direct contact with infected persons, or by contact with contaminated objects and surfaces [9–11]. The airborne transmission might be possible in specific settings in which procedures that generate aerosols were performed [12]. The clinical spectrum of COVID-19 could range from asymptomatic infection or mild upper respiratory tract illness to severe interstitial pneumonia with respiratory failure. The common symptoms were fever or chills, cough, shortness of breath, fatigue, muscle aches, headache, loss of taste or smell, sore throat, runny nose, nausea or vomiting, and diarrhea [1]. During the pre-symptomatic period, some infected persons could be contagious and therefore, people infected with COVID-19 could transmit the virus before significant symptoms developed [13, 14].

Certain comorbidities such as diabetes, heart diseases, chronic kidney disease, and obesity were strongly related to COVID-19 hospitalization and severity. Some studies demonstrated that older age, underlying diseases (including hypertension, diabetes, and cardiovascular disease, chronic lung disease, chronic kidney disease, and cancer) and presenting symptoms (including fever, cough, diarrhea, and breathlessness) might be predictors for the poor prognosis or severity in COVID-19 patients [15–18]. Older people with underlying medical problems like cardiovascular disease, diabetes, chronic respiratory disease, and cancer were more at risk of developing a serious illness and required referral for intensive care due to their low immune status [1, 19–24].

Myanmar, one of the tropical countries, administratively consists of seven regions, seven states and one Union Territory. Nay Pyi Taw, the capital, was designated as Union Territory. The total population was estimated at 54 million in 2020, with about 70% of the population resided in rural areas [25]. Lower respiratory tract infection and tuberculosis comprised the major causes of death in Myanmar [26]. Moreover, non-communicable diseases were estimated to account for 68% of all deaths, with an exposure to potential risk factors such as harmful use of alcohol, physical inactivity, tobacco use, raised blood pressure, and overweight or obesity. Cardiovascular diseases, diabetes mellitus, cancers and chronic respiratory diseases were reported to be the major contributing factors to the non-communicable diseases (NCDs) burden in Myanmar [27, 28].

In Myanmar, the first COVID-19 reported cases were identified on 23^rd^ March 2020 and there were 379 confirmed cases and 6 deaths during the first wave of epidemic. As the COVID-19 response in Myanmar, the National Level Central Committee on Prevention, Control and Treatment of COVID-19 established and announced the rules and regulations of COVID-19 preventive measures. To control the disease spread, the government implemented the mitigation measures as nonpharmaceutical interventions (NPIs) such as school closure, public education on handwashing, facemask wearing, social distancing, border closures, stay at home, partial lockdown, travel restriction, quarantined the people arrived back from abroad, contact tracing, early identification and hospitalization of all confirmed cases. The COVID-19 tests by reverse transcription polymerase chain reaction (RT-PCR) technology were being carried out at the National Health Laboratory in Yangon, Department of Medical Research (Lower Myanmar), Public Health Laboratories (Mandalay, Mawlamyaing, Taunggyi, and Muse), No. 1 Defence Services General Hospital (1000 bedded), No. 2 Defence Services General Hospital (1000 bedded), and No. 17 Military Hospital (100 bedded). The Ministry of Health and Sports prepared the Waibargi specialist hospital, South Okkalapa hospital and Phaunggyi COVID-19 treatment center in Yangon and the Kandaw Nadi hospital in Mandalay as designated hospitals for managing COVID-19 cases.

After almost a month without local transmission, the second wave started on 19th August 2020 in Rakhine State and the reported cases abruptly increased. The disease spread to whole country and Yangon became a major epicenter [29]. Myanmar had just 0.67 physicians per 1000 people in 2018 and 1.04 hospital beds per 1000 people in 2017 [30]. Moreover, there was 0.71 intensive care unit (ICU) bed and 0.46 ventilators per 100,000 populations in March 2020 [31]. To control the disease spread, the government performed further preventive and mitigation measures such as an increase in testing capacity by using rapid diagnostic test (Standard Q COVID-19 Antigen Test) in fever clinics and hospitals, expansion of quarantine facilities and treatment centers, restriction of public gatherings, suspension on international flights, closing restaurants and daycare facilities, enforcement of contact tracing, and hospitalization of all confirmed cases [32]. In the first week of October 2020, the government could conduct over 10,000 tests daily with more than 10% of test positivity rate. As of 14th March 2021, the total confirmed cases were 142,136 with 3201 deaths. The cases were increasingly reported in Yangon Region followed by Mandalay, Bago, Ayeyarwaddy, and Rakhine State [29, 32].

Although focusing on the public health measures like social distancing, the prohibition of public gatherings, and increased use of face masks to control the spread of COVID-19, these measures alone were uncertain to stop the pandemic due to the highly contagious nature of the disease. The continuation of learning more about the natural course of COVID-19, clinical presentation, comorbidity, and severity of disease was critical not only for healthcare system preparedness but also for clinical management or treatment [33, 34]. To better understand and adequately manage this novel threat, exploring detailed knowledge on appearance of symptoms and comorbidities in infected patients, and clarifying the severity were still required [35]. In Myanmar, there is no previous study regarding the severity of COVID-19 patients and its related factors. It is necessary to investigate them in Myanmar, where the epidemiological characteristics of COVID-19 disease may differ from other countries in demographics background, personal characteristics including genetics factors, climate, people's behavior, virus strains and NPIs. The aim of the study has therefore been to identify initial presenting symptoms, comorbidities, severity of disease and the associated factors of severe pneumonia among COVID-19 patients in Myanmar.

## Methods

### Study design and population

A cross-sectional study was conducted among COVID-19 patients attending at Hmawbi and Indine treatment centers from November to December 2020. All inpatients with confirmed SARS-CoV-2 infection by a positive result on RT-PCR testing of a nasopharyngeal sample, from two treatment centers, were included in this study.

### Study area

**Fig. 1:**
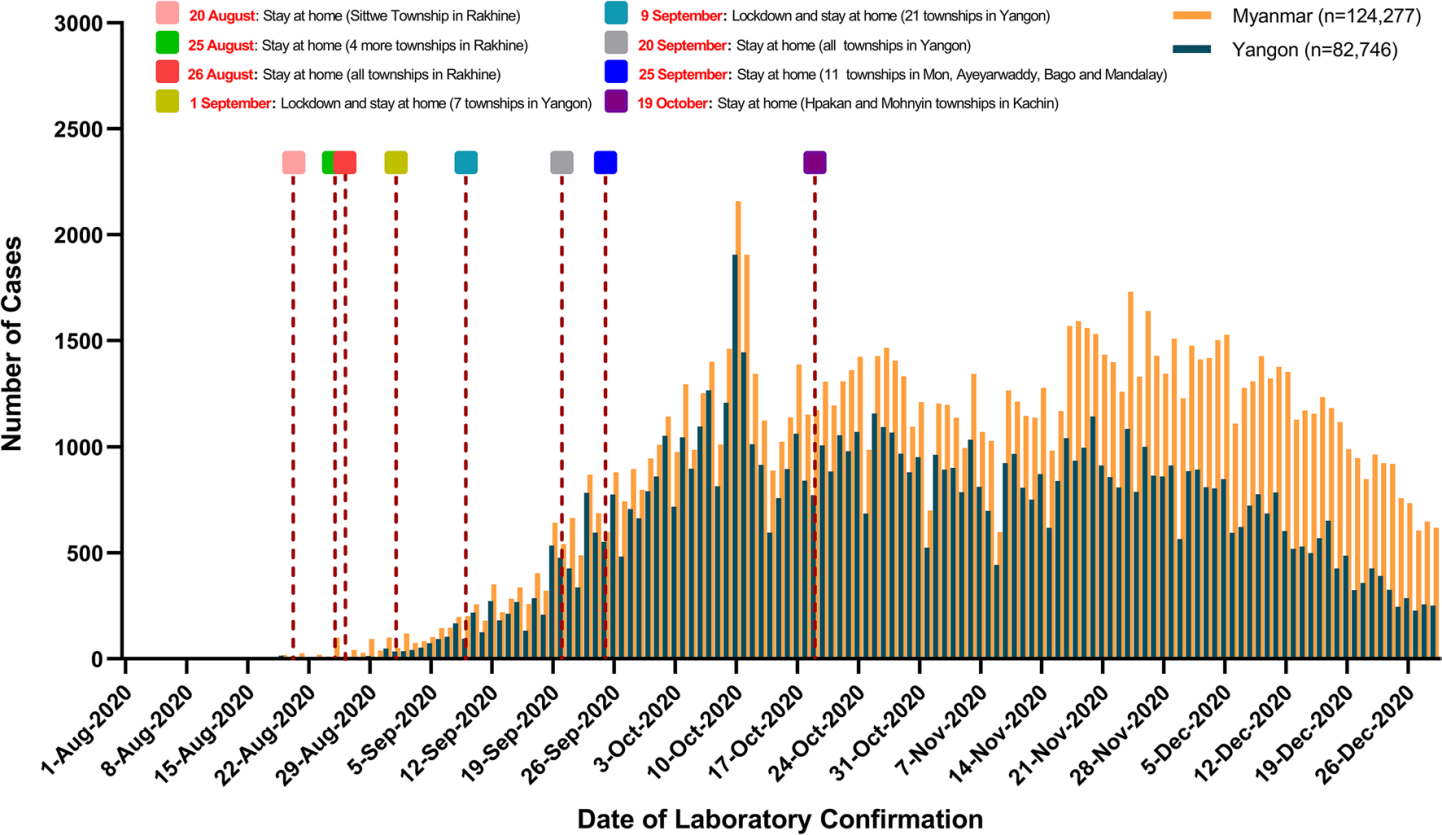
Daily confirmed COVID-19 cases in Myanmar and Yangon Region from August to December, 2020. The orange and blue bars represent the daily confirmed COVID-19 cases in Myanmar and Yangon Region, respectively. The dates of NPIs during second wave of epidemic are shown in different colors of rectangles. The total confirmed cases are 124,277 in Myanmar and 82,746 in Yangon Region from 1st August to 31st December 2020

The study was carried out at two purposively selected treatment centers, Hmawbi (270 bedded) and Indine (100 bedded) treatment centers, which were designated for confirmed COVID-19 patients and established in the second wave of the COVID-19 epidemic in Myanmar. The treatment centers were located in Hamwbi and Hlegu Townships, Northern District, Yangon Region, Myanmar. The daily COVID-19 confirmed cases in the whole country and Yangon Region from August to December 2020 are shown in Fig. 1. The patients who has tested positive for COVID-19 (both asymptomatic and symptomatic patients) in fever clinics, quarantine centers, and hospitals, mainly from Northern District, Yangon Region, were isolated and treated in Hmawbi and Indine treatment centers. The patient admission was determined by geographical background and hospital bed capacity. The treatment centers provided medical care and closely monitored the patients.

### Sample size determination and sampling technique

The sample size was calculated using a single population proportion formula [36] with an assumption of 95% confidence interval (CI), 5% margin of error, and 13.5% of severe COVID-19 patients [37]. Based on this assumption, the estimated minimum required sample size including 10% non-response rate was 197. All confirmed COVID-19 patients at two treatment centers during the study period (*n* = 222) were finally selected.

### Operational definitions

Body mass index (BMI) was a person’s weight in kilograms divided by the square of height in meters and it an indicator of body fatness. BMI was categorized as underweight (< 18.5 kg/m^2^), normal weight (18.5 to 24.9 kg/m^2^), overweight (25.0 to 29.9 kg/m^2^) and (≥ 30.0 kg/m^2^) obese. For the patients who were younger than 20 years, it was classified by the BMI-for-age weight status categories such as underweight (< 5th percentile), normal weight (5th to < 85th percentile), overweight (85th to < 95th percentile) and obesity (≥ 95th percentile) [38]. Current smoking was defined as an adult who has smoked 100 cigarettes in his or her lifetime and who currently smokes cigarettes. Alcohol drinking was defined as an adult who took at least 12 drinks in the past year but 3 drinks or fewer per week, on average over the past year. A contact person was defined as a person who experienced any one of the following exposures during the 2 days before and the 14 days after the onset of symptoms of a probable or confirmed case: face-to-face contact with a probable or confirmed case within 1 m and for more than 15 min, direct physical contact with a probable or confirmed case, and direct care for a patient with probable or confirmed COVID-19 disease without using proper personal protective equipment [39].

Comorbidity was a presence of more or additional medical conditions or diseases in COVID-19 patients. The initial presenting symptom was a symptom or group of symptoms about which the COVID-19 patient initially complains at the time of diagnosis (such as fever, muscle ache, cough, sore throat, dyspnea, etc.) and it was categorized as asymptomatic and symptomatic. The severity of COVID-19 was classified as mild, moderate and severe disease. Mild disease was symptomatic patients without evidence of viral pneumonia or hypoxia. Moderate disease was confirmed patients with clinical signs of pneumonia (fever, cough, dyspnea, and fast breathing). Severe disease was confirmed patient with clinical signs of pneumonia (fever, cough, dyspnea, and fast breathing) adding one of the following: respiratory rate > 30 breaths per min, severe respiratory distress and SpO_2_ < 93% on room air [39]. For the final analysis, the severity was categorized as not severe (asymptomatic, mild, and moderate) and severe disease.

### Data collection and procedure

Demographic characteristics (sex, age, state and region, district, education, occupation, height, weight, smoking status, alcohol drinking, contact with known COVID-19 cases, travelling history to foreign countries, and travelling history to townships under stay at home order), initial presenting symptoms (asymptomatic and symptomatic patients with fever, chills, difficulty in breathing, fatigue, muscle aches, headache, loss of smell, loss of taste, sore throat, runny nose, nausea or vomiting, diarrhea), comorbidity (hypertension, diabetes mellitus, coronary heart disease, chronic respiratory disease, cerebrovascular accident, cirrhosis of liver, hepatitis B virus infection, hepatitis C virus infection, chronic kidney disease, hematological disease, and cancer) and severity of disease (mild, moderate, and severe) were collected using a standardized case report form. The data were checked by two medical officers and then, supervision, completeness, and consistency of collected data were performed by the principle investigator.

### Statistical analysis

The collected data were entered into Microsoft Excel 2016 and exported to IBM SPSS Statistics for Windows, Version 23.0 (Armonk, NY: IBM Corp) for analysis. Descriptive statistics were presented as frequency and percentages for categorical variables and mean (standard deviation, SD) for continuous variables. Bivariable logistic regression analysis was performed to assess the relative impact of the predictor variables on the outcome variable. To control for potential confounding factors, multivariable logistic regression analysis was performed. All independent factors with *p* value < 0.05 in bivariable regression analysis were candidates for the multivariable logistic regression model. The results of group comparisons of risk factors and severity of disease were expressed as adjusted odds ratio (AOR) with 95% CI and a *p* value was set at < 0.05 for statistical significance.

## Results

A total of 222 COVID-19 patients were included in this study. Table 1 shows the demographic characteristics of COVID-19 patients. Among the total, 114 (51.4%) were females and 108 (48.6%) were males. The mean (± SD) age was 44.81 (± 16.99) years with a range of 17–86 years and 178 (80.2%) patients were younger than 60 years. Most of the patients, 210 (94.6%), were from Yangon Region and among them, 153 (72.9%) patients were from Northern District. In total, 99 (44.6%) patients had a high school education level and 130 (58.6%) patients were employed. In BMI of the patients, 131 (59.0%) were normal weight, 57 (25.7%) were overweight and 19 (8.6%) were obesity. Thirty-two (14.4%) patients were current smokers and 17 (7.7%) had a history of alcohol drinking. Overall, 98 (44.1%) patients had a history of contact with confirmed cases. Only two (0.9%) and eight (3.6%) patients travelled to foreign countries and townships under stay at home order, respectively.

**Table 1:** Demographic characteristics of COVID-19 patients (*n* = 222)

Variables	Frequency (%)
Sex	
Male	108 (48.6)
Female	114 (51.4)
Age (year)	
< 60	178 (80.2)
≥ 60	44 (19.8)
Mean ± SD; 44.81 ± 16.99 years, minimum 17 years, maximum 86 years	
State and region	
Yangon	210 (94.6)
Bago	5 (2.3)
Rakhine	3 (1.4)
Shan	3 (1.4)
Mon	1 (0.5)
District in Yangon Region (*n* = 210)	
Eastern District	19 (9.0)
Western District	26 (12.4)
Southern District	12 (5.7)
Northern District	153 (72.9)
Education	
Primary school education level	6 (2.7)
Middle school education level	32 (14.4)
High school education level	99 (44.6)
University and graduate	85 (38.3)
Occupation	
Employed	130 (58.6)
Unemployed	92 (41.4)
BMI (kg/m^2^)	
Underweight	15 (6.8)
Normal weight	131 (59.0)
Overweight	57 (25.7)
Obesity	19 (8.6)
Mean ± SD; 23.81 ± 4.26, Minimum 14.2, Maximum 36.8	
Smoking status	
No	190 (85.6)
Yes	32 (14.4)
Alcohol drinking	
No	205 (92.3)
Yes	17 (7.7)
Contact history	
No	124 (55.9)
Yes	98 (44.1)
Travel history to foreign countries	
No	220 (99.1)
Yes	2 (0.9)
Travel history to townships under stay at home order	
No	214 (96.4)
Yes	8 (3.6)

The initial presenting symptoms and comorbidities of COVID-19 patients are described in Table 2. Of all patients, 181 (81.5%) were symptomatic and among them, the most common initial presenting symptoms were fever 98 (54.1%) followed by loss of smell 91 (50.3%), cough 56 (30.9%), muscle ache 53 (29.3%), and headache 52 (28.7%). Eighty-four (37.8%) patients had comorbidities and among them, the most common comorbidities were hypertension 49 (58.3%), diabetes mellitus 25 (29.8%) and heart disease 22 (26.2%). As a severity 46 (20.7%) patients present with severe pneumonia (Table 3). Supplemental oxygen by nasal cannula or face mask was administered in 72 (32.4%) patients. All the patients with severe pneumonia, 46 (20.7%), were transferred to ICU and among them, there were 18 (39.1%) patients with high flow oxygen therapy, one patient (2.2%) received mechanical ventilation and six (13.0%) patients died.

**Table 2:** Initial presenting symptoms and comorbidities of COVID-19 patients

Variables	Frequency (%)
Initial presenting symptom (*n* = 222)	
Asymptomatic	41 (18.5)
Symptomatic	181 (81.5)
Presenting symptoms (*n* = 181, multiple response)	
Fever	98 (54.1)
Loss of smell	91 (50.3)
Cough	56 (30.9)
Muscle ache	53 (29.3)
Headache	52 (28.7)
Malaise	41 (22.7)
Dyspnea	40 (22.1)
Sneezing	37 (20.4)
Loss of taste	29 (16.0)
Sore throat	27 (14.9)
Chill	22 (12.2)
Diarrhea	22 (12.2)
Nausea and vomiting	14 (7.7)
Comorbidity (*n* = 222)	
No	138 (62.2)
Yes	84 (37.8)
Comorbid diseases (*n* = 84, multiple response)	
Hypertension	49 (58.3)
Diabetes mellitus	25 (29.8)
Heart disease	22 (26.2)
Hematological disease	10 (11.9)
Stroke	4 (4.8)
Chronic lungs disease	4 (4.8)
Hepatitis B virus infection	3 (3.6)
Hepatitis C virus infection	2 (2.4)
Cirrhosis of liver	1 (1.2)
Cancer	1 (1.2)

**Table 3:** Severity of COVID-19 patients (*n* = 222)

Variables	Frequency (%)
Asymptomatic	41 (18.5)
Mild	94 (42.3)
Moderate	41 (18.5)
Severe	46 (20.7)

In bivariable logistic regression analysis, the patients who were 60 years and older (COR: 3.71, 95% CI 1.79–7.65), those with overweight or obesity (COR: 5.29, 95% CI 2.65–10.60), those who were current smokers (COR: 8.82, 95% CI 3.91–19.93), those who were alcohol drinkers (COR: 2.98, 95% CI 1.07–8.32) and those who were with comorbidities (COR: 2.11, 95% CI 1.09–4.07) were more likely to develop severe pneumonia (Table 4). In multivariable logistic regression analysis, the factors such as age, BMI, and current smoking remained as the significant associated factors of severe disease. Aged 60 years and older patients were 2.88 times more likely to get severe pneumonia than the younger patients (AOR: 2.88, 95% CI 1.14–7.29). The patients with overweight or obesity were 3.87 times more likely to develop severe pneumonia than the patients with the counterpart (AOR: 3.87, 95% CI 1.80–8.33). The current smokers were 6.74 times more likely to get severe pneumonia compared to the non-smokers (AOR: 6.74, 95% CI 2.72–16.75).

**Table 4:** Association of severity of disease with demographic characteristics, comorbidity and initial presenting symptoms in COVID-19 patients

Variables	Severity of disease^a^		*p* value	COR (95% CI)	*p* value	AOR ^b^ (95% CI)
	Not severe *n* (%)	Severe *n* (%)				
Sex						
Male	89 (82.4)	19 (17.6)		1		
Female	87 (76.3)	27 (23.7)	0.264	1.45 (0.75–2.80)		
Age						
< 60 years	150 (84.3)	28 (15.7)		1		1
≥ 60 years	26 (59.1)	18 (40.9)	< 0.001	3.71 (1.79–7.65)	0.026	2.88 (1.14–7.29)
Education^c^						
Below university	111 (81.0)	26 (19.0)		1		
University and graduate	65 (76.5)	20 (23.5)	0.417	1.31 (0.68–2.54)		
Occupation						
Employed	105 (80.8)	25 (19.2)		1		
Unemployed	71 (77.2)	21 (22.8)	0.515	1.24 (0.65–2.39)		
BMI^d^						
Underweight or normal weight	130 (89.0)	16 (11.0)		1		1
Overweight or obesity	46 (60.5)	30 (39.5)	< 0.001	5.29 (2.65–10.60)	0.001	3.87 (1.80–8.33)
Smoking status						
No	163 (85.8)	27 (14.2)		1		1
Yes	13 (40.6)	19 (59.4)	< 0.001	8.82 (3.91–19.93)	< 0.001	6.74 (2.72–16.75)
Alcohol drinking						
No	166 (81.0)	39 (19.0)		1		1
Yes	10 (58.8)	7 (41.2)	0.037	2.98 (1.07–8.32)	0.430	1.68 (0.46–6.05)
Comorbidity						
No	116 (84.1)	22 (15.9)		1		1
Yes	60 (71.4)	24 (28.6)	0.026	2.11 (1.09–4.07)	0.782	1.13 (0.48–2.61)
Initial presenting symptoms						
Asymptomatic	41 (100.0)	0 (0.0)				
Symptomatic	135 (74.6)	46 (25.4)				

^a^Severity of disease was categorized as “not severe” (asymptomatic, mild and moderate) and “severe”

^b^All independent variables with *p* value < 0.05 in bivariable analysis were included in multivariable logistic regression model

^c^Education was categorized as “below university” (primary, middle and high school education level) and “university and graduate”

^d^BMI was classified as “underweight or normal weight” and “overweight or obesity”

## Discussion

The COVID-19 mainly affects the respiratory system, and some patients required intensive care due to a rapid progression of hypoxia, pneumonia, and acute respiratory distress syndrome. This study investigated the prevalence of symptomatic, comorbidities, severity, and the associated factors of severity in COVID-19 patients. In this study, the female patients were more than the males and it was in line with the studies done in the USA [18] and Germany [40]. However, males were more infected than the females in the studies done in Thailand [37], Singapore [41] and China [42]. There was no difference of sex distribution in the studies conducted in China [23, 43]. The recent study reported that 19.8% of patients were the older age and it was higher than the findings obtained in the China study 12.3% [23] and Thailand study 11.9% [37]. Nonetheless, it was lower than the findings of studies conducted in the USA 28.3% [18] and Germany 26.7% [40]. More than one-third, 34.3%, of the patients were overweight or obesity in this study and it was higher than the result of Thailand study 32.9% [37]. Conversely, it was lower than the findings of studies done in Germany 38.2% [40] and China 47.5% [44]. The reason for these variations might be due to the differences in the socio-economic and geographical nature of the study areas, variation in sample size, and distinction in lifestyle factors.

The prevalence of symptomatic patients (81.5%) in the current study was lower than studies done in Thailand 94.8% [37] and Korea 91.3% [45], but higher than in the China study 70.6% [23]. This inconsistency might be due to variation of diagnostic and hospitalization criteria of COVID-19 patients. A substantial number of undocumented infections leading to no symptoms might enable the rapid spread of SARS-CoV-2 [46]. The explanations for the detection of nearly 20.0% of the asymptomatic patients in this study might be due to well achievement in case finding, close contact tracing, and massive surveillance of probable and suspected COVID-19 cases. In Myanmar, the government expanded the testing capacity for primary contacts and imported cases as a priority of testing. In addition, hospitalization of all COVID-19 patients including symptomatic and asymptomatic might control the ongoing community spread of SAR-CoV-2. In the current study, it was surprising that loss of smell, apart from fever and cough, was one of the most common symptoms, and this finding was contrary to that of previous studies done in the health centers and hospitals reported that fatigue, sore throat, shortness of breath and rhinorrhea were the most common presenting symptoms [18, 37, 44, 45, 47].

In this study, 37.8% of the patients had comorbid diseases and it was higher than the findings of the studies conducted in China 15.8% [23], Thailand 25.0% [37] and Singapore 28.3% [41]. This discrepancy could be attributed to variation in the prevalence of chronic diseases across age, gender distribution, and geographic region. NCDs were identified as a priority public health problem in Myanmar and cardiovascular disease was one of the NCDs with the highest impact on mortality [28]. Over 2012 to 2017, most of the admitted patients with NCDs were middle and older aged population with the median and interquartile range of 39 (25–55) years and 51.6% of those were males [48]. In the current study, hypertension and diabetes mellitus were the most common comorbidities and these results supported the findings of the earlier studies conducted in hospitalized COVID-19 patients [18, 23, 40, 42, 45, 47].

As the severity of COVID-19, the prevalence of severe pneumonia was 20.7% and it was higher than the results of studies done in China (9.1% [23] and 7.2% [43]), Thailand (13.5%) [37], and Singapore (16.7%) [41]. It was lower than the finding of study done in China stated that 35.6% of the COVID-19 patients developed severe pneumonia [42]. It was possible that these results were due to variability of chest imaging findings (chest radiograph or CT), the difference in criteria of patient isolation and hospitalization, and contrast to protocol and management guidelines of COVID-19 patients. The symptomatic patients tend to have severe inflammation in the lungs, which more commonly leads to disease progression. The symptomatic patients had a higher risk of developing bilateral pneumonia and less likely to show improvement of pneumonia than asymptomatic patients [23].

In the recent study, age was a significant determinant of severe pneumonia in COVID-19 patients and it might be explained by the fact that older people were particularly susceptible to develop more infections as natural immunity declined gradually at older ages. Another explanation for this finding could be that the older people might have more expression of ACE2 encoded by the *ACE2* gene and have other conventional factors such as reduced immunity, poor organ function, or coexisting comorbid diseases which might have increased risk of disease severity [49]. This finding was keeping with the previous studies done in Thailand [37] and China [43, 44, 47] reported that older age was a potential predictive factor of progression to severe pneumonia in SARS-CoV-2 infected patients.

The immune function and response in viral infections were influenced by lifestyle factors, overweight and obesity. This study confirmed that overweight or obesity was associated with the development of severe pneumonia in COVID-19 patients. The possible reason might be due to the fact that people with overweight or obesity might have comorbidities including metabolic diseases, cardiovascular diseases, and cancers that were susceptible to infection. Moreover, they had a significantly large amount of ACE2 receptor in adipose tissue and were more likely to be infected with SARS-CoV-2, which resulted in increased viral shedding, immune inactivation, and cytokine storm [50]. This finding also supported the evidence from other studies conducted in Thailand [37] and Germany [40] reported that overweight or obesity patients were more likely to get severe pneumonia than normal weight patients.

Tobacco contains components that disrupt the normal epithelial lining of the respiratory system leading to increased oxidative injury and impairment of mucociliary clearance [51]. Smoking was also a significant predictor of pneumonia in COVID-19 patients in the current study. This could be because tobacco smoke suppressed the function of innate immune cells, including respiratory epithelium, alveolar surfactant, macrophage, neutrophils, and lymphocytes. This could make smokers were more susceptible to develop the complications of COVID-19, such as pneumonia. This result matched those observed in earlier studies done in China [52, 53] and Turkey [54] reported that there was an association between the current smoking status and disease severity of COVID-19. However, this finding was contrary to previous studies which had evidence that smoking was not associated with the severity of COVID-19 [45, 47, 55].

There were some limitations in this study. Firstly, it was relatively difficult to establish a causal relationship between severity and independent variables due to the cross-sectional nature of this study. A longitudinal study with a larger sample size could be applied to find out the higher strength of association. Secondly, although the results were representative of the population with the same demographic characteristics, further research using a random sampling method should be conducted to have a more representative cohort. Thirdly, the asymptomatic patients might have developed symptoms after collecting the data and they could be over looked. Lastly, some hematological and biochemical markers (such as white blood cell count, hemoglobin, C-reactive protein, erythrocyte sedimentation rate, alanine transaminase, aspartate transaminase, prothrombin time, D-dimer, etc.) that might be related with severity of disease were not measured in this study.

## Conclusions

In a hospital-based study in Myanmar, approximately one-fifth of asymptomatic patients with test positive for COVID-19 were identified, and therefore, screening, surveillance and contact tracing should be more expanded for early detection of asymptomatic people and rapid control of community spread. The prevalence of initial presenting symptom, comorbidity and severe pneumonia in COVID-19 patients were 81.5%, 37.8% and 20.7%, respectively. Loss of smell was one of the common presenting symptoms in these treatment centers. This study reapproved the association of demographic factors and lifestyle factors with the severity of COVID-19. Therefore, the healthcare providers should pay particular attention to the COVID-19 patients who were aged 60 years and older, overweight or obesity, and current smokers to detect and reduce the disease severity as early as possible.

## Data Availability

The data analyzed for this manuscript are available from the corresponding author and can be made accessible upon reasonable request.

## Abbreviations

ACE 2: Angiotensin-converting enzyme 2
AOR: Adjusted odds ratio
BMI: Body mass index
CI: Confidence interval
COR: Crude odds ratio
COVID-19: Coronavirus disease 2019
ICU: Intensive Care Unit
NCDs: Non-communicable diseases
NPIs: Nonpharmaceutical Interventions
RT-PCR: Reverse transcription polymerase chain reaction
SARS-CoV-2: Severe Acute Respiratory Syndrome Coronavirus 2
SD: Standard deviation
WHO: World Health Organization
